# Hbs and Rst adhesion molecules provide a regional code that regulates cell elimination during epithelial remodeling

**DOI:** 10.1016/j.isci.2026.114971

**Published:** 2026-02-18

**Authors:** Miguel Ferreira-Pinto, Mario Aguilar-Aragón, Christa Rhiner, Eduardo Moreno

**Affiliations:** 1 Champalimaud Foundation, Lisbon, Portugal

**Keywords:** Biological sciences, Cell biology, Molecular biology

## Abstract

Cellular interactions and mechanical forces are fundamental in shaping epithelial tissue architecture. In the *Drosophila* notum, tissue compression at the midline promotes epithelial cell elimination. Here, we conducted a multi-step RNAi screen and identified 47 diverse regulators of notum epithelial remodeling. We find that the two cell adhesion proteins Hibris (Hbs) and Roughest (Rst) show high expression in zones of cell survival versus low levels in areas of cell pruning. Notum-wide knock-down of *hbs* or *rst* or homogenous *hbs* overexpression disrupts cell death patterns and results in adult tissue malformations. Local suppression of Hbs and Rst in Hbs^high^/Rst^high^ territories triggers ectopic cell elimination indicating that Hbs/Rst can instruct cell removal. Interestingly, Hbs but not Rst is regulated by compaction-sensitive EGFR signaling, positioning Hbs as an integrator of both mechanical and cell property cues. These findings uncover a novel adhesive landscape that shapes the thorax midline and potentially other organs.

## Highlights

• Multi-step screen identifies new regulators of notum epithelial remodeling
• Hibris and Roughest specify an adhesive landscape required for adult notum formation
• Local changes in Hibris/Roughest expression can instruct cell elimination
• Hibris, but not Roughest is regulated by mechano-sensitive EGFR signaling

## Graphical abstract

**Figure:**
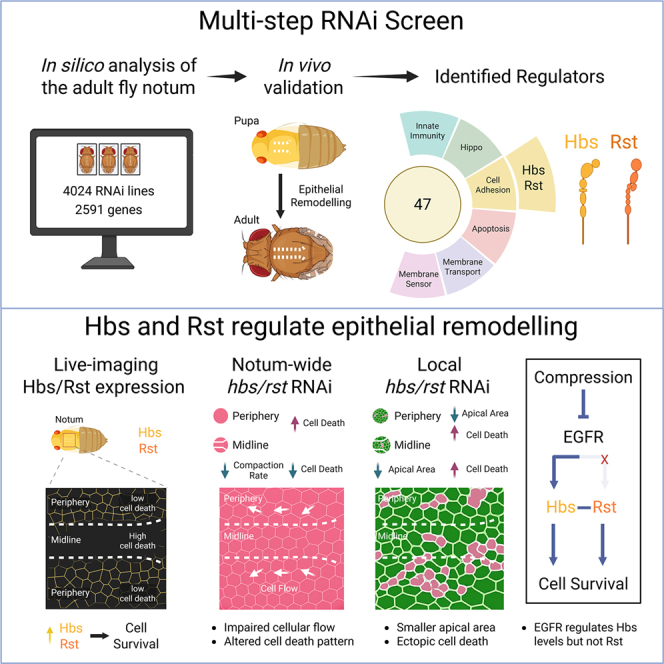

## Introduction

All animals, regardless of their different sizes and shapes, develop based on fundamental rules that guide how tissues grow and organize. Epithelial tissues establish the body’s fundamental architecture, forming sheets and tubes that give rise to organs such as the gut, lungs, and skin. A finely tuned balance between cell division, cell death, cell migration, and cell rearrangement ensures the precise tissue shape.^1^ Mechanical forces drive crucial aspects of morphogenesis, such as elongation or folding,^2^ cell-cell interactions,^3^^,^^4^^,^^5^ and adjustment of cell death rates.^6^^,^^7^^,^^8^ Compression forces, in particular, have been shown to act as an integral-feedback mechanism regulating the elimination of compacted suboptimal cells in epithelial co-cultures of MDCK cells, zebrafish epidermal fin edges and the *Drosophila* notum, an epithelium giving rise to the adult dorsal thorax structure.^7^^,^^9^^,^^10^^,^^11^^,^^12^

In zebrafish, mechanical crowding engages the stretch-activating channel Piezo1 and sphingosine 1-phosphate signaling, which converges on Rho-kinase-dependent myosin contraction promoting cell extrusion.^9^^,^^10^ As a cell intrinsic factor, high levels of P53 have been shown to be sufficient to induce crowding hypersensitivity in MDCK co-cultures.^9^^,^^13^ In *Drosophila*, cell elimination in the notum is also regulated by apoptotic components, requiring the induction of the pro-apoptotic factor *head involution defective* (*hid*) followed by caspase activation and microtubule disassembly, which precede cell extrusion.^11^^,^^12^^,^^14^

Upstream of *hid* induction, the downregulation of compaction-induced EGFR/ERK survival signaling has been shown to be important for cell death at the notum midline.^11^ Nevertheless, how EGFR senses mechanical compression^11^^,^^15^ and which other components are required to guide cell selection upstream of pro-apoptotic factors has remained unclear. Candidate genes mediating fitness-dependent cell pruning such as *flower*,^16^ *azot*,^17^ and JNK^18^^,^^19^ did not control cell elimination in the notum^11^^,^^12^ suggesting that other—yet unknown factors—regulate cell pruning and notum remodeling.

Here, we conducted an extensive 2-step genetic screen in the well-characterized *Drosophila* notum and identified a novel set of modulators that shape epithelial restructuring. The top regulators include the genes *hibris* (*hbs*) and *roughest* (*rst*). These two Immunoglobulin-like cell adhesion molecules have been previously found to act as recognition module-proteins during the formation of the highly organized fly retina^20^^,^^21^^,^^22^ and the patterning of wing sensory organs.^23^^,^^24^^,^^25^ In the retina, the heterophilic binding of Hbs and Rst maximizes the cell-cell contacts of a subset of interommatidial progenitor cells (IPS), while excess IPS with insufficient adhesion are eliminated.^20^ Similarly, selective adhesion conferred by Hbs and Rst on different cell types are thought to mediate cell sorting into the precise zigzag patterns of *Drosophila* wing margin hairs.^23^

How the two cell adhesion molecules could regulate highly dynamic tissue movements in a developing epithelial sheet is not known and their link to mechanical forces remains unexplored. Here, we uncover a novel role of Hbs and Rst in specifying an adhesive landscape during epithelial remodeling in the fly notum, which—when perturbed—leads to malformation in adult tissues. We show that relative levels of Hbs and Rst are sufficient to instruct cell elimination versus cell survival, functioning as a cell selection code that guides proper organization of epithelial cells in the developing notum. We further find that one component of the Hbs/Rst surface code is modulated by compaction-sensitive EGFR/ERK signaling. Our findings put forward a new framework, in which Hbs and Rst constitute new players of the regulatory network, potentially integrating both EGFR/ERK-dependent and independent signals to control epithelium architecture.

## Results and discussion

### A multi-step screen to identify novel epithelial remodeling regulators

**Figure 1:**
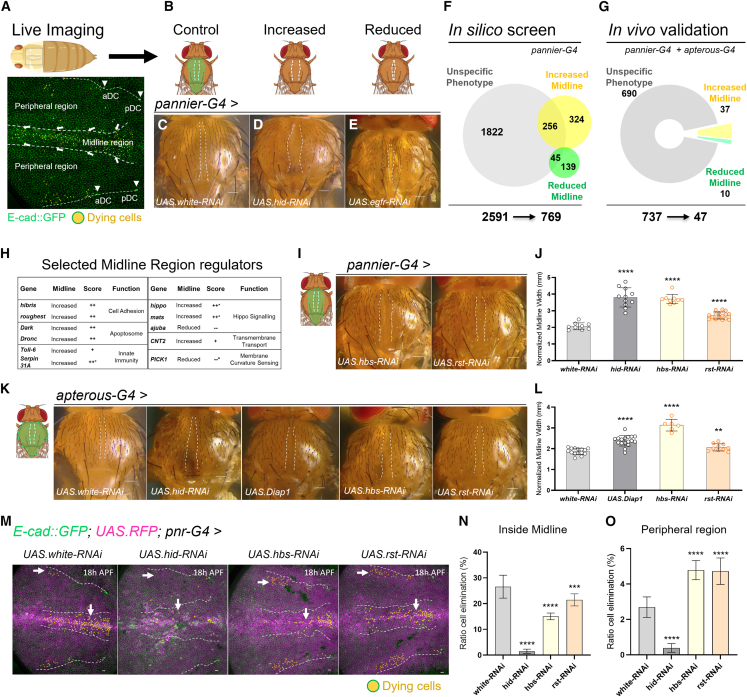
A multi-step screen identified Hbs and Rst as novel epithelial remodeling regulators (A) Schematic of the notum epithelium during pupal development. The central bristle row marks the midline (white dashed lines). Arrows indicate convergence cell flow toward the midline. Dying cells are shown in yellow. E-cadherin (green) labels cell membranes for tracking cell fate over time. The aDC/pDC macrochaetae delimit the peripheral region of the notum. (B) Schematic illustrating different midline phenotypes (white dashed lines). The *pannier* domain is marked (green). (C–E) Adult notum images showing midlines of *pannier-Gal4* driven *white* RNAi (control), *hid* RNAi and *egfr* RNAi. White dashed lines delineate the midline. Scale bars: 10 μm. (F) Recovered midline phenotypes in the “*in silico* screen” based on RNAi activation with *pannier-Gal4*. Overlapping areas indicate genes with both midline-specific and nonspecific phenotypes. (G) Midline phenotypes assessed by *in vivo* gene knock-down driven by *pannier* and *apterous-Gal4* notum drivers. (H) Table highlighting selected midline regulators, validated *in vivo*. Scores denote ++ strong midline increase; + intermediate midline increase; −− strong midline decrease; − intermediate midline decrease. Asterisks mark genes that caused mild notum defects outside the midline. (I) Adult notum images showing increased midline width upon *hbs* and *rst* RNAi in the pannier domain. Scale bars: 10 μm. (J) Quantification of normalized midline width (mm) upon gene downregulation using *pannier-Gal4*. Each dot represents one adult fly thorax. Statistical significance was assessed by unpaired *t* test or Mann-Whitney against the control; ∗∗∗∗*p* < 0.0001. Error bars represent standard deviation (SD). (K) Adult notum images displaying midline phenotypes of control (*UAS.white* RNAi), *UAS.hid* RNAi, *UAS.Diap1*, *UAS.hbs* RNAi and *UAS.rst* RNAi driven by *apterous Gal-4*. Scale bars: 10 μm. (L) Quantification of normalized midline width (mm) based on RNAi driven by *apterous-Gal4*. Each dot represents one adult fly thorax. Statistical significance was assessed by unpaired *t* test or Mann-Whitney against the control; ∗∗∗∗*p* < 0.0001. Error bars represent standard deviation (SD). (M) Z-projections of live pupal nota at 18 h APF (700 min movies) with quantified cell death in control flies (*white* RNAi) and upon *hid* RNAi, *hbs* RNAi, and *rst* RNAi. Yellow cells indicate future dying cells within the *pannier* domain (RFP, magenta). White arrows point to cell delamination events. Cell membrane is marked with ubi-EcadGFP (green). White dashed lines mark the midline. Peripheral regions analyzed are delimited by the aDC and pDC bristles (white dashed lines). Scale bars: 10 μm. (N and O) Quantification of cell delamination events (ratio) inside the midline region and in the periphery over 12 h (700 min, from 18 h to 30 h APF). Error bars represent the standard error of the mean (SEM) for each condition. Sample sizes: *white* RNAi (control): *n* = 4 nota, 1,531 cells (midline), 4,816 cells (outside); *hid* RNAi: *n* = 2 nota, 1,618 cells (midline), 3,148 cells (outside); *hbs* RNAi: *n* = 4 nota, 1,848 cells (midline), 5,118 cells (outside); *rst* RNAi: *n* = 4 nota, 2,071 cells (midline), 5,066 cells (outside). Statistical analysis was performed using Fisher’s exact test against the control; ∗∗∗∗*p* < 0.0001).

During pupal notum development, cells are eliminated in highly reproducible patterns in the notum, ensuring the correct notum size and shape in adult flies (Figure 1A). As previously reported, cell elimination events are more frequent inside the midline region of the notum than outside due to increased cell crowding at the midline (Figure 1A).^11^^,^^12^ The notum midline cells give rise to the central section of the adult thorax (Figures 1B and 1C). The silencing of key cell death regulators in the developing notum has been shown to affect the width of the midline region in the adult thorax.^12^ We tested silencing of the pro-apoptotic gene *hid*, which resulted in an increased midline, whereas suppression of the negative cell death regulator EGFR led to a reduced midline (Figures 1D and 1E). Based on these results, we concluded that the adult midline phenotype can serve as a reliable readout for identifying novel regulators of cell death.

To uncover new components mediating epithelial remodeling, we devised a two-step genetic screen taking advantage of the midline phenotype. First, we conducted an *in silico* screen, in which we analyzed midlines in a vast collection of adult fly notum images from the public bristle screen database (BSD), based on a genome-wide RNAi screen performed by the Knoblich Lab^26^ (Figure S1A). We quantified midline phenotypes in all available BSD pictures, corresponding to 4024 RNAi lines covering 2591 genes and placed them into three different categories. Genes, for which the RNAi resulted in an altered midline width—without causing major notum defects—were classified as “increased midline” or “reduced midline” (Figures 1F and S1B). Genes, for which silencing affected the overall notum morphology were excluded and categorized as “unspecific”, alongside genes that did not cause midline change (Figures 1F and S1B). Although this approach may have led to the exclusion of midline regulators that also disrupt other developmental processes, it still resulted in 769 retained candidate regulators, for which the strength of the observed midline phenotype was recorded, e.g., strongly (++) to moderately increased (+) or strongly (−−) to moderately reduced (−) (Figure S1B and Table S1). All genes for which at least one RNAi line gave rise to a significant midline phenotype were included in the *in silico* candidates (Figure 1F). Based on RNAi line availability, 95.8% of these genes were next tested in a rigorous *in vivo* screen (Figure 1G). Two independent RNAi lines per gene were analyzed in combination with the notum drivers *pannier-Gal4* and *apterous-Gal4*, which show slightly different expression in the pupal notum (Figures S1C–S1H). As a result, this multi-step screen led to the identification of 47 high confidence candidates for epithelial remodeling at the pupal midline (Figures 1G and S1I). The midline phenotypes for all 47 candidates are shown in Table S1 and Figure S2.

Table S1. Screening results - Characterization of novel regulators of epithelial remodeling

Among the 47 identified regulators of cell remodeling at the midline, with 37 showing increased and 10 reduced midline width, we found the apoptosome proteins Dark and Dronc, innate immunity components *toll-6* and *serpin31A*, Hippo signaling factors (*hippo*, *mats*, and *ajuba*), the transmembrane transporter CNT2, the membrane curvature sensor PICK1 and the cell adhesion molecules Hibris and Roughest (Figure 1H). Pro-apoptotic genes were expected to be recovered in the approach as caspase activation was previously shown to control cell elimination in the pupal notum.^11^^,^^12^ Additionally, the screen revealed numerous novel regulators with a wide range of functions, including transcription factors and cell-tension regulators (Figures S1J and S1K), whose contributions to epithelial remodeling can be explored in the future.

### Hibris and roughest regulate cell pruning in the notum

Cell adhesion is an important quality to transmit mechanical forces across a tissue or mediate specific cell-cell recognition. We therefore focused on the two transmembrane proteins Hbs and Rst. Hbs/Rst have been previously found to regulate cell rearrangement during *Drosophila* eye development^20^ and sensory organ patterning in the wing.^23^^,^^24^^,^^25^ We found that RNAi of *hbs* or *rst* consistently resulted in an increased midline width for both tested RNAi lines and for both *pannier* and *apterous* notum drivers (Figures 1I–1L). Hbs suppression caused a similarly increased midline as RNAi of *hid* or overexpression of the *Drosophila* inhibitor of caspases (Diap1), which block apoptosis (Figures 1J–1L). The midline increase with *rst* RNAi was lower than with *hbs*, but still highly significant (Figures 1I–1L). *kirre* and *sns*, which have been found to interact with *hbs* or *rst* in other contexts, were not among the “*in silico*” candidates, but when tested *in vivo*, did not appear to cause significant midline phenotypes (Figure S1L).

To further study the role of Hbs and Rst, we quantified cell elimination events using a live imaging set-up for the pupal notum, consisting of a glass window inserted into the pupal case, which allows recording of cell dynamics over 700 min across developmental timepoints from 18 to 30 h after pupa formation (APF) (Figure 1A). In this context, the term “cell elimination” refers to extrusion of apoptotic midline cells, a process previously shown to require *hid* activation and caspase activity.^11^^,^^12^ The presence of *pannier*-driven nuclear RFP and presence of membrane GFP (E-cadherin::GFP) facilitated the tracking of cell elimination in combination with RNAi (Figure 1M). Notum-wide knock-down of *hbs* or *rst* with *pannier-Gal4* reduced cell death at the midline compared to the control (RNAi of *white*) (Figures 1M and 1N; Videos S1 and S2). The effect was less strong compared to *hid* RNAi, which almost completely blocked cell death during the imaged time window. In contrast, pannier-driven *hbs or rst* knock-down led to elevated cell elimination at the notum periphery (Figures 1M and 1O).

Video S1. Cell death events during notum epithelium remodelling, related to Figure 1 Z-projections of live pupal nota at 18 h APF (700 min movies) with quantified cell death in control flies (*white* RNAi - Video S1). Yellow cells indicate future dying cells within the *pannier* domain (RFP, magenta). Cell membrane is marked with *ubi-Ecad::GFP* (green). White lines mark the midline region and delimit the peripheral region near the aDC and pDC bristles. Scale bars: 10 μm.

Video S2. Hbs is necessary during notum epithelium remodelling, related to Figure 1 Z-projections of live pupal nota at 18 h APF (700 min movies) with quantified cell death upon *hbs* RNAi (Video S2). Yellow cells indicate future dying cells within the *pannier* domain (RFP, magenta). Cell membrane is marked with *ubi-Ecad::GFP* (green). White lines mark the midline region and delimit the peripheral region near the aDC and pDC bristles. Scale bars: 10 μm.

Together, the findings demonstrate that Hbs and Rst are required to establish the local pattern of cell pruning at the remodeling notum midline and correct formation of adult dorsal thorax structures, potentially by shaping mechanical forces or cell intercalation dynamics.

### Hbs and Rst shape global epithelial remodeling

Since *hbs* and *rst* RNAi resulted in increased cell elimination at the notum periphery, we hypothesized that Rst/Hbs expression could be predictive of areas of cell survival in the notum, potentially by reducing mechanical stress or facilitating pro-survival signaling.

To test this, we recorded Hbs and Rst localization in the developing notum in real time, taking advantage of knock-in reporter lines that allow faithful monitoring of Hbs and Rst as EGFP-tagged fusion proteins.^27^ We confirmed that Hbs::EGFP and Rst::EGFP were expressed in the characteristic patterns at the dorsal-ventral (DV) boundary and in the notum region of the wing disc, as previously shown for Hbs/Rst antibodies^24^^,^^25^ (Figures S3A–S3D).

**Figure 2:**
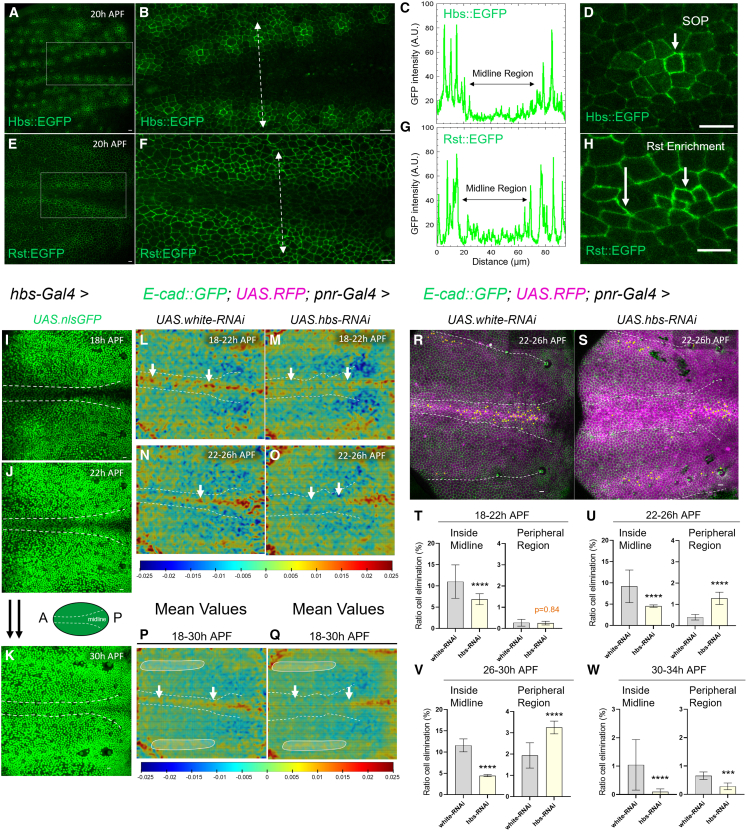
Hbs and Rst drive global and local epithelial remodeling (A–H) Z-projections of live pupal nota at 20 h APF expressing the HbsEGFP (A) or RstEGFP reporter (E). Close-up views of the midline region (outlined by white rectangles in A and E) are shown for Hbs (B) and Rst (F). Intensity profiles of Hbs (C) and Rst (G) measured along the white dashed line crossing the midline region. Close-up views showing Hbs localization in sensory organ precursors (SOP) (D, arrow), and differential Rst enrichment in notum cells (H, arrows). Scale bars: 10 μm. (I–K) Z-projections of live pupal nota at 18 h (I), 22 h (J), and 30 h (K) APF from 700 min movies expressing GFP (green) in the Hbs domain. The midline region is highlighted in white. Scale bars: 10 μm. Schematic shows the notum orientation (Anterior–Posterior) and the midline region. (L–Q) Averaged PIV vector fields showing tissue convergence rates from live imaging movies of control (*white* RNAi) and *hbs* RNAi pupae over matched time intervals. For *white* RNAi: 18–22 h APF (L), 22–26 h APF (N), and average from 18 to 30 h APF (P). For *hbs* RNAi: 18–22 h APF (M), 22–26 h APF (O), and average from 18 to 30 h APF (Q). Red regions indicate high convergence; blue regions indicate low convergence. White arrows highlight reduced cell convergence in the midline region upon *hbs* RNAi compared to control. Circles highlight increased convergence in the periphery upon *hbs* RNAi. (R and S) Z-projections of live pupal nota at 22 h APF from 700 min movies used to quantify cell death from 22 to 26 h APF in control flies (*white* RNAi) and upon *hbs* RNAi. Yellow cells indicate future dying cells within the pannier domain (RFP, magenta) and Ubi-EcadGFP (green). White dashed lines mark the midline. Scale bars: 10 μm. (T–W) Quantification of cell delamination events (ratio) inside midline and in the periphery region across sequential developmental intervals. Time windows: 18–22 h APF (T), 22–26 h APF (U), 26–30 h APF (V), 30–34 h APF (X). Error bars represent standard error of the mean (SEM). Statistical analysis was performed using Fisher’s exact test against the control; ∗∗∗∗*p* < 0.0001.

We then started notum recordings at 18–20 APF and noted that both Hbs::EGFP and Rst::EGFP were strikingly absent from the forming midline and enriched in cells located at the periphery (Figures 2A–2G). This pattern was highly consistent and quantification of signal across the notum yielded a clear minimum of Hbs and Rst signal in the area of cell elimination (midline) (Figures 2C and 2G). However, their distribution outside the midline was not identical: Hbs appeared strongly expressed in notum sensory organ precursors (SOPs), in line with reported Hbs expression in bristle cells,^24^^,^^25^ with decreasing levels the farther cells were located from the central SOP (Figures 2B and 2D). Rst expression was more uniform in peripheral areas but appeared often selectively enriched in certain cell junctions compared to others (Figures 2F and 2H).

In order to unequivocally correlate Hbs levels to one cell and track its fate in areas of differential cell compaction, we next imaged Hbs dynamics in flies, in which *hbs-Gal4* drives nuclear GFP (*UAS.nlsGFP*). As previously observed with the reporter lines, *hbs-Gal4*-driven GFP appeared low in midline cells from 18 to 22h APF, only reaching equal expression to peripheral cells by 30 h APF (Figures 2I–2K). We could not perform similar experiments for Rst due to the unavailability of suitable *rst-Gal4 lines*.

It is known that during the earlier stages of pupal notum formation, high cell compaction and cell elimination rates at the midline contrast with moderate cell compaction and low cell elimination in peripheral regions.^11^^,^^12^ We speculated that the detected increase of peripheral cell elimination, caused by notum-wide Hbs/Rst suppression, could arise from globally altered mechanical forces across the notum.

To test this, we employed particle image velocimetry (PIV) to track tissue dynamics in nota with widely suppressed Hbs expression (*pnrG4*>*hbs* RNAi) versus controls (*white* RNAi) (Figures 2L–2Q). The generated compaction rate maps (calculated as divergence of the vector field) showed that when Hbs^high^ and Hbs^low^ territories were abolished due to RNAi, the midline compression zone failed to build up efficiently and the cell strain rate at the midline was decreased at 18–22 h APF (Figures 2L, 2M; Figures S3E and S3F). Lower cell compaction at the anterior half of the midline also persisted at later time points (22–26 h APF) (Figures 2N, 2O; Figures S3E and S3F). When we integrated the total tissue deformation over time, the plots confirmed the lack of a midline compaction in *hbs* knock-down conditions and showed more prominent areas of moderate compression in peripheral regions (Figures 2P and 2Q, circled areas). Similar alternations in notum compaction (reduced in the center, increased at periphery) was detected upon *rst* RNAi (Figures S3G–S3I). This suggests that peripheral Hbs and Rst levels are needed to drive cellular flow toward the midline. The direct involvement of Hbs in notum remodeling was supported by the fact that Hbs^low^ expression in the center strongly correlated with high cell compaction, whereas Hbs^high^ levels in the periphery overlapped with areas of low or moderate compaction (Figures 2I and 2J vs. Figures 2L and 2N).

To better understand the link between Hbs expression, mechanical forces and cell elimination, we analyzed the impact of notum-wide *hbs* RNAi on cell death in short time windows (Figures 2R and 2S). During the first 4 h of pupal formation, suppressed Hbs levels caused a reduction in cell delamination rates at the midline (Figure 2T) without altering cell death in the periphery. From 22 h on, the clear reduction of cell pruning at the midline continued, combined with ectopic cell death in the periphery, potentially causing a “flow” to the peripheral region, as cells accumulate at the widening midline (Figure 2U) in *hbs* RNAi flies. In the later stages of notum development, *hbs* knock-down caused both reduced cell pruning at the midline and increased peripheral cell death (Figure 2V). Finally, cell elimination comes to a halt when Hbs expression turns uniform at the end of notum remodeling (30 h APF) (Figures 2K and 2W). We also recorded nota with *pnr*-driven *rst* RNAi, which similarly showed increased cell elimination at the notum periphery (Figures S3J–S3M).

These findings suggest that Hbs and Rst expression in the peripheral notum is necessary to establish a well-positioned central compression zone that likely acts as a driving force for mechano-sensitive cell elimination.

### Hbs/Rst guide local cell selection at the midline

**Figure 3:**
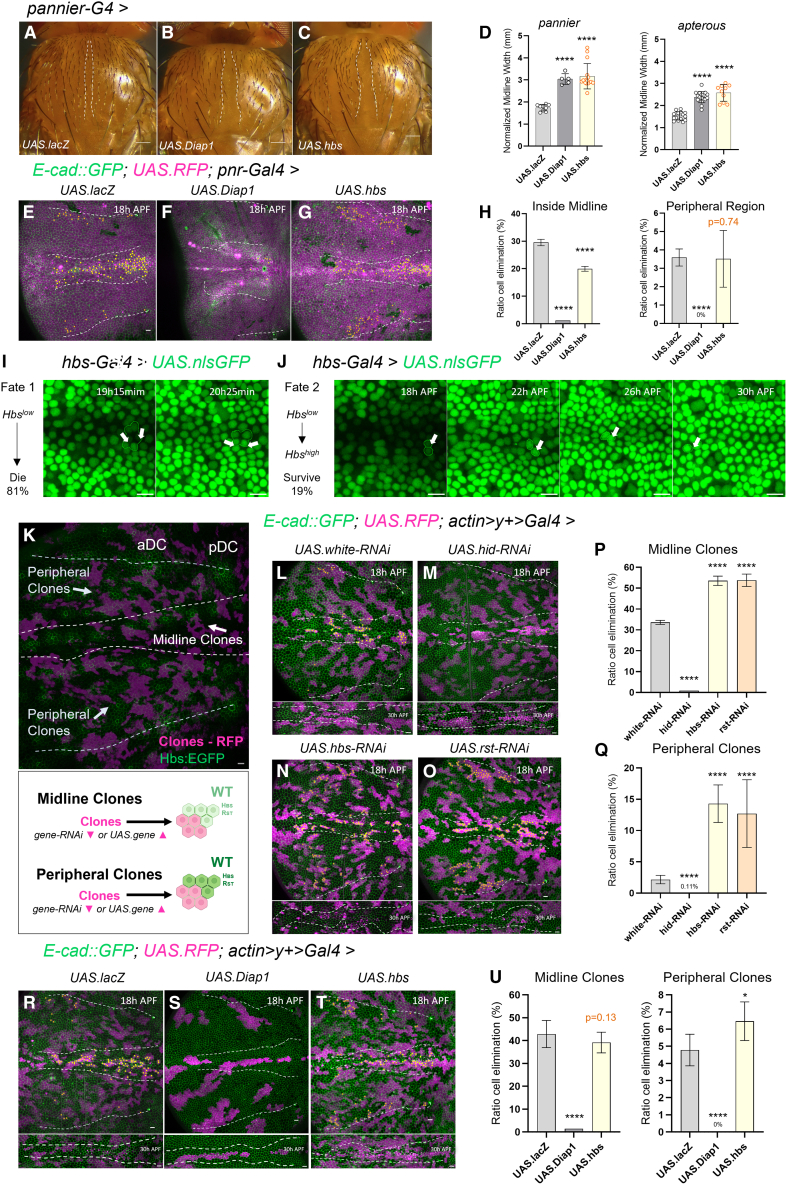
Hbs and Rst instruct cell elimination to fine-tune cell selection in the notum (A–C) Adult notum images displaying midline phenotypes of control (*UAS.lacZ*), *UAS.Diap1* and *UAS.hbs* using *pannier-Gal4*. Scale bars: 10 μm. (D) Quantification of normalized midline width (mm) upon gene overexpression using *pannier-Gal4*. Each dot represents one adult fly thorax. Statistical significance was assessed by unpaired *t* test or Mann-Whitney against the control (∗∗∗∗*p* < 10^−4^). Error bars represent standard deviation (SD). (E–G) Z-projections of live pupal nota at 18 h APF (700 min movies) with quantified cell death in control flies (*UAS.lacZ*) and upon *Diap1* and *hbs* overexpression. Yellow cells indicate future dying cells within the pannier domain (RFP, magenta). Cell membranes are labeled with *ubi-Ecad::GFP* (green). The midline region is outlined with white dashed lines. Peripheral regions analyzed are delimited by the aDC and pDC bristles (white dashed lines). Scale bars: 10 μm. (H) Quantification of cell delamination events (ratio) inside the midline region (left) and in the periphery (right) over 12 h (700 min; 18–30 h APF). Error bars represent the standard error of the mean (SEM). Sample sizes: *UAS.lacZ*: *n* = 3 nota, 1,167 cells (midline), 3,802 cells (outside); *UAS.Diap1*: *n* = 1 notum, 1,035 cells (midline), 1,784 cells (outside); *UAS.hbs*: *n* = 2 nota, 829 cells (midline), 2,836 cells (outside). Statistical analysis was performed using Fisher’s exact test against the control; ∗∗∗∗*p* < 10^−4^. (I–J) Close-up views of two distinct cell fates in the pupal notum midline region during epithelium remodeling. Fate 1 (I) – Low Hbs-expressing cells elimination (arrows indicate delaminating cells). Fate 2 (J) – Low Hbs-expressing cells (white arrows) dynamically upregulate Hbs over time. Scale bars: 10 μm. (K) Schematic illustrating two distinct cell interaction scenarios following clone induction in the pupal notum, based on the endogenous expression pattern of Hbs. Peripheral clones downregulating *hbs* interact with wild-type (WT) cells expressing high Hbs levels, while midline clones face WT cells with low Hbs. Conversely, *hbs*-overexpressing clones compete against high-Hbs WT cells in the periphery and low-Hbs WT cells in the midline. (L–O) Z-projections of live pupal nota at 18 h APF (700 min movies) with quantified cell death in control flies (*white* RNAi) and upon *hid* RNAi, *hbs* RNAi and *rst* RNAi. Yellow cells mark future dying cells within clones (RFP, magenta). Cell membranes are labeled with *ubi-Ecad::GFP* (green). Midline regions are outlined by white dashed lines at 18 h APF and again at 30 h APF below each image. Peripheral regions analyzed are delimited by the aDC and pDC bristles (white dashed lines). Scale bars: 10 μm. (P–Q) Quantification of cell delamination events (ratio) in midline clones and peripheral clones over 12 h (700 min; 18–30 h APF). Error bars represent SEM. Sample sizes: *white* RNAi: *n* = 3 nota, 469 cells (midline), 1,989 cells (outside); *hid* RNAi: *n* = 1 notum, 246 cells (midline), 942 cells (outside); *hbs* RNAi: *n* = 4 nota, 503 cells (midline), 2,385 cells (outside); *rst* RNAi: *n* = 2 nota, 289 cells (midline), 1,277 cells (outside). Statistical significance was assessed using Fisher’s exact test against the control; ∗∗∗∗*p* < 10^−4^. (R–T) Z-projections of live pupal nota at 18h APF (700min movies) with quantified cell death in control flies (*UAS.lacZ*) and upon *Diap1* and *hbs* overexpression. Yellow cells indicate future dying cells within clones (RFP, magenta). Cell membranes are labeled with *ubi-Ecad::GFP* (green). White dashed lines mark the midline region at 18 h and 30 h APF. Peripheral regions analyzed are delimited by the aDC and pDC bristles (white dashed lines). Scale bars: 10 μm. (U) Quantification of cell delamination events (ratio) in midline clones (top) and peripheral clones (bottom) over 12 h (700 min; 18–30 h APF). Error bars represent SEM. Sample sizes: *UAS.lacZ*: *n* = 3 nota, 411 cells (midline), 2,024 cells (outside); *UAS.Diap1*: *n* = 1 notum, 70 cells (midline), 231 cells (outside); *UAS.hbs*: *n* = 4 nota, 481 cells (midline), 2,345 cells (outside). Statistical significance was assessed using Fisher’s exact test against the control; ∗∗∗∗*p* < 10^−4^.

Since abrogating Hbs/Rst territories by RNAi resulted in midline width defects, we next asked if homogenous Hbs overexpression would interfere with midline formation. Indeed, quantification of the adult notum midline in flies with *pannier-Gal4-*dependent or *apterous-Gal*4-dependent Hbs overexpression (*UAS.hbs*) resulted in an increased midline, similar to overexpression of Diap1, which blocks apoptosis (Figures 3A–3D). Interestingly, live imaging of the pupal notum showed that Hbs overexpression reduced the ratio of cell elimination at the midline compared to controls (*UAS.lacZ*), while cell death at the periphery remained unchanged (Figures 3E–3H), suggesting that providing increased Hbs levels to midline cells prevented their elimination.

To evaluate this idea, we studied unmanipulated nota that build pronounced compression zones at the midline and correlated cell fate of midline cells with their initially presented levels of Hbs expression. We found that cells with very low Hbs signal tended to be eliminated (81% of 50 tracked cells), especially when located between higher Hbs-expressing cells (Figure 3I and Video S3). Still, 19% of Hbs^Low^ cells persisted and this fate was associated with gradual Hbs upregulation around 22 h APF (Figure 3J and Video S4). Overall, the absence of Hbs expression at the midline was highly predictive of cell elimination, whereas Hbs expression favored cell maintenance. This observation bears resemblance to fly retina patterning, during which interommatidial cells with high Hbs/Rst interactions are kept, compared to Hbs^low^ counterparts that are eliminated.^20^

Video S3. Low Hbs expressing cells are eliminated during notum epithelium remodelling, related to Figure 2 Close-up view of low Hbs-expressing cells elimination inside the midline region during live pupal notum imaging. Arrows point to dying cells. Scale bars: 10 μm.

Video S4. Low Hbs expressing cells can upregulate Hbs to survive during notum epithelium remodelling, related to Figure 2 Close-up view of low Hbs-expressing cells inside the midline region gradually upregulating Hbs over time during live pupal notum imaging. Cells gradually upregulating Hbs are circled at 60-minute intervals to highlight progressive Hbs expression. Scale bar: 10 μm.

To understand if relative differences in the Hbs and Rst code would be sufficient to alter cell fate, we created RFP-marked *hbs* and *rst* RNAi clones surrounded by wild-type (wt) notum cells (RFP negative) using a heat-shock inducible flippase, together with an *actin>stop>Gal4* flip-out cassette. The random flip-out produced RNAi clones within the midline and in the periphery (Figure 3K). We then quantified cell elimination ratios in *hid* RNAi, *hbs* RNAi and *rst* RNAi clones versus control clones (*white* RNAi) in the Hbs/Rst^low^ midline or in the Hbs/Rst^high^ periphery (Figures 3L–3Q; Videos S5 and S6).

Video S5. Cell death events in clones during notum epithelium remodelling, related to Figure 3 Z-projections of live pupal nota at 18 h APF (700 min movies) with quantified cell death in control flies (*white* RNAi – Video S5). Yellow cells mark future dying cells within clones (RFP, magenta). Cell membranes are labelled with *ubi-Ecad::GFP* (green). Midline regions are outlined by white dashed lines at 18h APF. Peripheral regions analysed are delimited by the aDC and pDC bristles (white peripheral lines). All cells analysed are circled at the onset of the movie. Scale bars: 10 μm.

Video S6. Hbs and Rst direct cell elimination to fine-tune cell selection during notum epithelium remodelling, related to Figure 3 Z-projections of live pupal nota at 18 h APF (700 min movies) with quantified cell death upon *hbs* RNAi (Video S6). Yellow cells mark future dying cells within clones (RFP, magenta). Cell membranes are labelled with *ubi-Ecad::GFP* (green). Midline regions are outlined by white dashed lines at 18 h APF. Peripheral regions analysed are delimited by the aDC and pDC bristles (white peripheral lines). All cells analysed are circled at the onset of the movie. Scale bars: 10 μm.

We found that knock-down of Hbs or Rst in midline clones increased cell elimination compared to control midline clones (*hbs* RNAi: +20.1%; *rst* RNAi; +18.82%) (Figure 3P). This suggested that normally low Hbs levels of midline cells still contributed to survival as further depletion by RNAi increased their pruning. In comparison, *hid* RNAi clones showed no cell death as expected. Interestingly, mosaic midlines (*hbs or rst* RNAi vs. wt cells) all developed a meandering midline by 30 h APF, which was not observed for *hid* RNAi *mosaic* midlines (Figures 3L–3O) suggesting that heterogeneous Hbs/Rst levels and altered cell pruning impacted cell rearrangement to form the midline structure. In the notum periphery, local *hbs* and *rst* knock-down in clones provoked a significantly increased cell elimination rate (*hbs* RNAi: +12.18%; *rst* RNAi*:* +10.68%) (Figure 3Q) in areas where cell elimination is normally rare indicating that Hbs/Rst protect against cell pruning. The ectopic cell death in the periphery observed with clonal manipulation was clearly higher than rates seen with notum-wide RNAi (Figure 1O), pointing to a cell non-autonomous effect arising from relative differences in Hbs levels between manipulated and neighboring wild-type cells.

Next, we tested clonal overexpressing of Hbs, Diap1, or control β-galactosidase in the midline versus the notum borders (Figures 3R–3U). Given that cells upregulating Hbs may persist at the midline (Figure 3J), we expected that high Hbs levels may render midline cells more resistant to cell elimination. However, we found that midline clones with activated Hbs overexpression showed similar elimination rates as controls, whereas Diap1-overexpression provided full protection (Figure 3U). This suggested that cells showing very high Hbs expression are not selected for maintenance and more physiologic levels may be required. In turn, Hbs overexpression in peripheral clones caused slightly increased cell elimination (+1.68%) (Figure 3U) indicating that overexpression slightly impacted survival. This may be due to an elevated cell mixing index, which has been described for Hbs-overexpressing clones in the wing disc, which adopted more jagged conformations.^28^ It therefore appears that Hbs levels among neighboring cells need to be highly in sync to not distort cell cohesive properties.

Altogether our results support that Hbs and Rst function on one hand at the tissue scale, to modulate cellular flow and tissue compaction (Figure 2) and on the other hand locally, to fine-tune cell selection at the midline (Figure 3). In the compression-exposed midline, the Hbs/Rst-modulated pruning may serve to retain cells with adequate shape and adhesive properties.

### Differential regulation of Hbs and Rst by EGFR/ERK signaling

**Figure 4:**
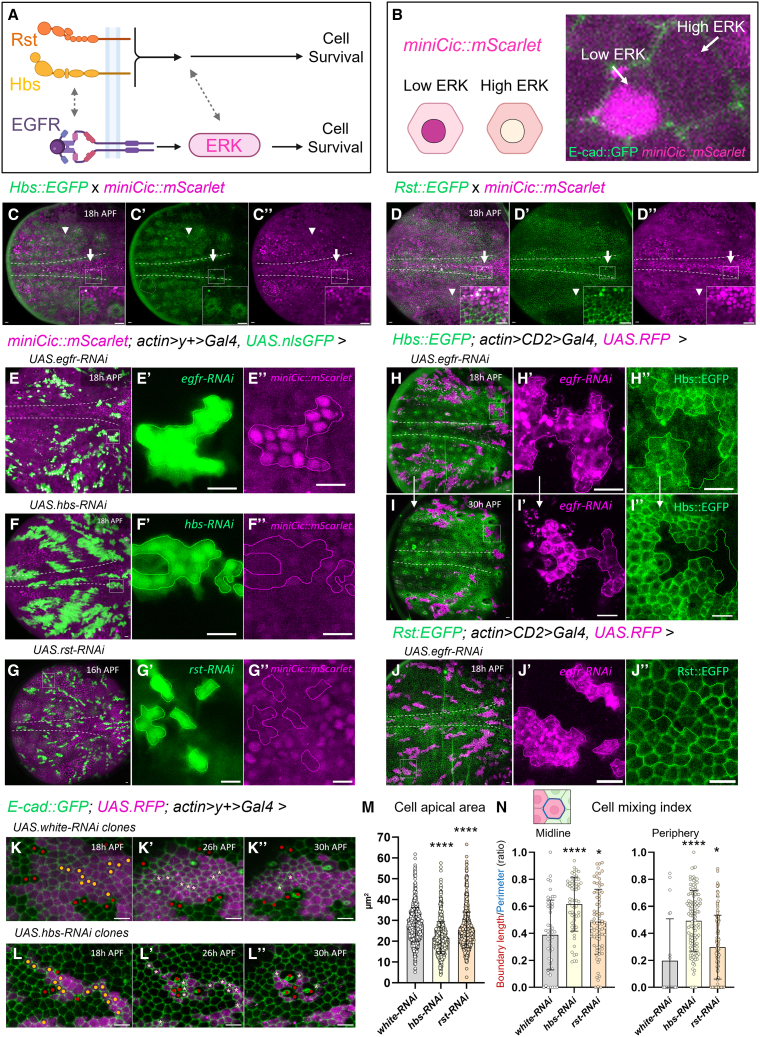
Hbs and Rst expression patterns largely correlate with EGFR/ERK activity but are differentially regulated by EGFR/ERK signaling (A) Schematic model summarizing the proposed roles and potential interactions between hibris (Hbs), roughest (Rst), and the Egfr/Erk signaling pathway during notum epithelium remodeling. (B) Schematic illustrating miniCicmScarlet reporter activity (magenta), which inversely correlates with ERK signaling. High miniCic signal indicates low ERK activity; low miniCic signal reflects high ERK activation. (C and D) Z-projections of live pupal nota at 18 h APF expressing either HbsEGFP and RstEGFP reporters (green), together with the miniCicmScarlet reporter (magenta, inversely correlated with ERK activity). Close-up views highlight the correlation between Hbs, Rst and ERK activity inside and outside the midline region. White arrows highlight regions of low Hbs/Rst levels and low ERK activity and arrow heads regions of high Hbs/Rst levels and high ERK activity. Gray dashed circles indicate rare regions where Hbs and Rst expression levels appear to be inversely correlated with ERK activity. Scale bars: 10 μm. (E–G) Z-projections of live pupal nota at 16/18 h APF showing ERK activity (miniCicmScarlet, magenta) in clones (GFP, green) upon *egfr* RNAi, *hbs* RNAi, or *rst* RNAi. Close-up views of clone-containing regions (white rectangles) are shown to the right for each panel. Scale bars: 10 μm. (H–I) Z-projections of live pupal nota expressing the HbsEGFP reporter in the presence of Egfr knock-down clones (magenta) at 18 h and 30 h APF. Close-up views of the same clone-containing regions (white rectangles) are shown on the right. Scale bars: 10 μm. (J) Z-projection of live pupal notum at 18h APF expressing RstEGFP (green) and Egfr knock-down clones (magenta). Close-up view of a clone-containing region (white rectangle) is shown to the right. Scale bars: 10 μm. (K and L) Close-ups of control peripheral clones (*white* RNAi) and *hbs* RNAi peripheral clones from live pupal notum movies (Figures 3M and 3P). Images show the same clones over time at 18 h (K, L), 22 h (K′, L′), 26 h (K″, L″), and 30 h APF (K‴, L‴). Yellow clone cells at 18 h indicate future dying cells; red cells mark neighboring WT cells. Asterisks indicate clone cells moments before elimination. Scale bars: 10 μm. (M and N) Quantification of cell apical area (μm^2^) and cell mixing index (defined as the fraction of a cell’s perimeter is in contact with non-clonal wild-type neighbors across the clone boundary and calculated as boundary length (red on scheme)/perimeter (blue on scheme)) in control clones (*white* RNAi), in *hbs* and in *rst* RNAi clones inside the midline region and in the periphery at 18 h APF. Each dot represents one clone cell. Sample sizes: Cell apical area of all clone cells; *white* RNAi, *n* = 1659 cells; *hbs* RNAi, *n* = 1516 cells; *rst* RNAi, *n* = 1576 cells. Cell mixing index of future dying cells; *white* RNAi, *n* = 49 cells midline and *n* = 20 cells periphery; *hbs* RNAi, *n* = 60 cells midline, *n* = 116 cells periphery; *rst* RNAi, *n* = 77 cells midline, *n* = 136 cells periphery. Error bars represent standard deviation (SD). Statistical significance was determined using the Mann-Whitney test; ∗∗∗∗*p* < 0.0001 against control.

It has been previously shown that cell crowding at the midline induces downregulation of the EGFR/ERK pathway and upregulation of pro-apoptotic *hid*, leading to cell elimination.^11^ EGFR is known to be enriched in adherens junctions in the notum,^11^ similarly to what we detected for Hbs and Rst (Figure S3B), suggesting potential interactions among these membrane proteins (Figure 4A). To address this question, we utilized a live sensor of ERK activity (*miniCic::mScarlet*),^29^ which shifts from nuclear to cytoplasmic localization upon ERK phosphorylation.^11^ Accordingly, nuclear mScarlet (magenta) is detected in cells with low ERK activity, whereas cytoplasmic signal indicates high ERK activation (Figure 4B). When examining imaginal wing discs, we found that areas of low ERK activity correlated with sites of low Hbs and Rst expression (Figures S4A–S4C). When we imaged the early pupal notum, we similarly detected that low ERK activity at the midline and in small patches in the periphery, overlap with Hbs^low^ and Rst^low^ signal (Figures 4C and 4D, arrows). On the other hand, most peripheral areas with high ERK activity also showed high Hbs and Rst levels (arrowheads). By the end of notum development, the entire notum displayed high levels of ERK and widespread Hbs and Rst expression (Figure S4D).

To determine whether Hbs and Rst regulate the EGFR/ERK pathway, we assessed ERK activity in *hbs* or *rst* knock-down clones. *hbs* and *rst* RNAi cells could still activate ERK signaling, in contrast to *egfr* RNAi clones that showed inactive, nuclear ERK (Figures 4E–4G) indicating that Hbs and Rst do not function upstream of EGFR/ERK. To understand if EGFR/ERK may in turn regulate Hbs or Rst, we assessed levels in *egfr* knock-down clones. Interestingly, we found that suppression of EGFR caused an almost complete loss of Hbs membrane signal, in most cells analyzed at 18 h APF and 30 h APF (Figures 4H and 4I). As an exception, few peripheral clones spanning bristle cells retained Hbs expression, suggesting an alternative regulation of Hbs in bristle formation (Figure S4E). EGFR signaling has been previously shown to be downregulated in response to laser-induced tissue compaction in the notum.^11^ In line with these findings, we detected that compressive forces created in laser-induced wounds triggered Hbs::GFP downregulation (Figures S4F and S4G).

On the other hand, midline clones overexpressing EGFR or constitutively active *ras*^*V12*^^30^ induced Hbs expression, confirming Hbs dependency on EGFR signaling (Figures S4H and S4I). In contrast, Rst expression was not reduced by *egfr* RNAi (Figure 4J), showing that the EGFR/ERK pathway regulates Hbs but not Rst expression.

The pro-survival EGFR/ERK signaling has been shown to prevent the activation of pro-apoptotic *hid*, which promotes caspase activation and cell death.^11^ Given that EGFR/ERK signals are also required to maintain robust Hbs expression, it is conceivable that lower Hbs levels prepare cells for being compressed and extruded by reducing adhesion and membrane resistance to compression. In support of this, we found that clones with compromised Hbs or Rst levels were generally smaller and showed more uneven, notched outlines, often with cells dying at the clone border (yellow dots) (Figures 4K–4L). When quantified, cells in *hbs* and *rst* RNAi clones consistently displayed a smaller apical surface compared to controls (Figure 4M). Moreover, cells that later underwent cell death showed a higher mixing index compared to controls (Figure 4N).

These findings suggest that Hbs expression contributes to mechanical resistance whereas dynamic downregulation of Hbs in midline cells, potentially due to decreasing EGFR/ERK activity, may facilitate cell shrinkage and detachment. We found that Hbs showed normal downregulation in midline clones with blocked *hid* expression (Figure S4J) indicating that Hbs is regulated upstream of the recruitment of pro-apoptotic factors. Also, providing EGFR knock-down cells with high levels of Hbs did not rescue them from being eliminated (Figures S4K–S4P), likely because the activation of apoptotic fate *(hid* induction) and following caspase activation cascades cannot be reversed by improvement of cell quality aspects.

Despite Hbs sensitivity to tissue compression, recent findings have revealed a role for cell geometry independent of mechanical forces,^31^ where smaller cells compared to their neighbors were preferentially eliminated in a Notch or Hippo-dependent manner. As we recovered Hippo signaling components in the screen and there is also some evidence that Notch signaling can regulate Rst localization during pupal eye development,^32^ it is possible that the Hbs interaction partner Rst could be regulated by Notch or Hippo signals.

In summary, our findings reveal that the Hbs/Rst expression landscape in the notum is required to direct correct cell elimination. Apart from a local effect at the midline, we find that Hbs and Rst expression may dictate the pattern of cell death across the notum by affecting cell cohesion and compaction rate, enhancing our understanding of how organs are shaped.

In particular, Hbs emerges as a key regulator that guides cell dynamics at the tissue level and regulates final selection of midline cells exposed to mechanical stress. We propose that Hbs acts as an integrating platform to convey both mechanical signals from compaction-dependent competition via EGFR/ERK and likely other cell cohesive signals, potentially via heterophilic interaction with Rst.

Hbs and Rst are evolutionary conserved, with *NPSH1* (human *hbs*) and *KIRREL* (human *rst*) promoting specific cell-cell interactions essential for the formation of the three-dimensional epithelial glomerular podocyte architecture.^33^ The overexpression of NPSH1 has been found to drive cell aggregation in cancer cells^34^ and mutations in *KIRREL1* confer a proliferative advantage to several human cancer cell lines, in a mechanism dependent on cell density levels.^35^ These findings raise the possibility that Hbs/Rst patterns may shape cell behavior and survival not only during epithelial remodeling, but also as a consequence of epithelial renewal or in the course of cancer formation. Insight into the molecular mechanisms linking mechanical forces to cell adhesion via Hbs and Rst may therefore provide new avenues to restrict cancer growth in the future.

### Limitations of the study

Our *in silico* screen relied on available images in the bristle screen database from the pupal notum RNAi screen performed by Mummery-Widmer et al.^26^ Consequently, genes not included in that dataset were not assessed and additional regulators of notum remodeling may have been missed.

We pin-pointed that Hbs is regulated by the EGFR/Ras axis and could show that Hbs knock-down leads to striking changes in cell apical surface area and the increased mixing with wild-type cells. Nevertheless, the detailed downstream mechanism mediating these changes remains to be explored. In our preliminary results, we did not detect any obvious effects on actomyosin patterns or E-cadherin localization. Further work addressing the impact of altered cell adhesion on mechanical forces and/or signaling will show how the dynamic Hbs/Rst patterns shapes epithelial remodeling in detail.

## Resource availability

### Lead contact

• Requests for further information and resources should be directed to and will be fulfilled by the lead contact, Eduardo Moreno (eduardo.moreno@research.fchampalimaud.org).

### Materials availability

• This study did not generate new unique reagents.
• All screen analysis and data generated in this study are available from the lead contact without restriction.

### Data and code availability

#### Data

• This paper analyses existing, publicly available data, accessible at [DOI: https://doi.org/10.1038/nature07936 or Database: https://bristlescreen.imba.oeaw.ac.at/index.php]
• All data reported in this paper will be shared by the lead contact upon request.

#### Code

• This paper does not report original code.

#### Additional information

• Any additional information required to reanalyse the data reported in this paper is available from the lead contact upon request.

## Acknowledgments

We thank the Bloomington Stock Center and the VDRC Stock Center for fly stocks; Romain Levayer, António Jacinto, Florence Janody, Matthias Eggel for sharing antibodies or fly stocks. We sincerely thank Catarina Brás Pereira for discussions, advice and support in several stages of this work. We thank the Champalimaud Foundation’s Fly Facility and the ABBE Imaging Platform for excellent technical support, and Dr. Telmo Pereira (Nova Medical School) for assistance with the laser-ablation experiments.

Work in our laboratory was funded by Fundação D. Anna de Sommer Champalimaud e Dr. Carlos Montez Champalimaud, the 10.13039/501100000781European Research Council (Consolidator Grant to E.M.: Active Mechanisms of Cell Selection: From Cell Competition to Cell Fitness, 2014–2019; grant agreement ID 614964), the Portuguese Foundation for Science and Technology-FCT (PTDC/BIA-CEL/3594/2020—DOI 10.54499/PTDC/BIA-CEL/3594/2020). C.R. is funded by HR23-00860 from La Caixa & FCT, the ERC-Portugal program from FCT and Champalimaud Foundation. M.F-P. was funded by La Caixa (LCF/BQ/DR20/11790018—PhD Program in Biology—Doctoral INPhINIT—RETAINING 2020); Fly platform was funded by CONGENTO LISBOA-01-0145-FEDER-022170, co-financed by FCT (Portugal) and Lisboa 2020, under the PORTUGAL 2020 agreement (10.13039/501100008530European Regional Development Fund).

## Author contributions

E.M. conceived and designed the project. M.F.-P., M.A., C.R., and E.M. designed the experiments, M.F.-P. conducted the experiments and analyzed data; M.F.-P. and M.A. performed the multi-step screen data acquisition and analysis. M.A., C.R., and E.M. supervised the work. M.F.-P. and C.R. wrote the manuscript with contributions from the other authors.

## Declaration of interests

The authors declare no competing interests.

## STAR★Methods

### Key resources table

**Table:** 

REAGENT or RESOURCE	SOURCE	IDENTIFIER
**Experimental models: Organisms/strains**		

*Drosophila: UAS-lacZ (II)*	Bloomington Center	BDSC_8529
*Drosophila: UAS-luciferase*	Bloomington Center	BDSC_35788
*Drosophila: UAS-white dsRNA (III)*	Bloomington Center	BDSC_ 33623
*Drosophila: UAS-Diap1 (III)*	Bloomington Center	BDSC_6657
*Drosophila: UAS-hid dsRNA (III)*	VDRC	GD_8269
*Drosophila: apterous-Gal4 (II)*	F. Janody (Porto i3S)	–
*Drosophila: pannier-Gal4 (III)*	Bloomington Center	BDSC_3039
*Drosophila: UAS.Dicer2 (I);; pannier-Gal4 (III)*	Bloomington Center	BDSC_25758
*Drosophila: UAS.rst-RNAi (III)*	VDRC	GD_951 GD_27223
*Drosophila: UAS.hbs-RNAi (II)*	VDRC	GD_40898 KK_105913
*Drosophila: UAS.hbs (III)*	Bloomington Center	BDSC 41797
*Drosophila: hbs-Gal4*	Bloomington Center	BDSC 77720
*Drosophila: UAS.kirre-RNAi*	VDRC	GD_3111 GD_27227
*Drosophila: UAS.sns-RNAi*	VDRC	GD_877 KK_109442
*Drosophila: UAS.egfr (II)*	Bloomington Center	BDSC_5368
*Drosophila: UAS.egfr*^*WT*^*(III)*	Bloomington Center	BDSC_9535
*Drosophila: UAS.egfr-RNAi (II)*	VDRC	KK_107130
*Drosophila: UAS.egfr-RNAi (III)*	Bloomington Center	BDSC_31526
*Drosophila: Rst::EGFP (X)*	Bloomington Center	BDSC_59410
*Drosophila: Hbs::EGFP (II)*	Bloomington Center	BDSC_65321
*Drosophila: tubminiCic::mCherry (II)*	Moreno et al.^11^	–
*Drosophila: tubminiCic::mScarlet (II)*	Valon et al.^29^	–
*Drosophila: endo-Ecad::GFP, tubminiCic::mScarlet (II)*	Valon et al.^29^	–
*Drosophila: yw hs-FLP; Ecad::Tomato*	A. Jacinto (Lisbon NMS)	–
*Drosophila: yw hs-FLP; ubi-Ecad::GFP, UAS.mRFP; actin>y+>Gal4*	Moreno et al.^11^	–
*Drosophila: hs-FLP22; endo-Ecad::GFP, tub-Gal80ts; actin>CD2>Gal4, UAS.nlsRFP/TM6B*	R. Levayer (Paris Inst.Curie)	–
*Drosophila: W; If/CyO; actin>>Gal4, UAS.GFP/TM6B*	M. Eggel (Basel)	–
*Drosophila: W; actin>CD2>G4, UAS.RFP*	Lab collection	ID_379
*Drosophila: UAS-ras*^*V12*^*(III)*	Karim and Rubin^30^	–
*Drosophila: W; actin>CD2>G4, UAS.RFP; UAS.ras*^*V12*^	F. Janody (Porto i3S)	–
*Drosophila: If/CyO; 2x tub-Gal80*^*ts*^*/TM6B*	M. Moita (Lisbon Champalimaud Foundation)	–

**Software and algorithms**		

MATLAB with Image processing toolbox and Statistics and Machine learning toolbox	Mathworks	https://www.mathworks.com/
PIVlab	W. Thielicke and E. J. Stamhuis (2014)	http://www.PIVlab.de
Fiji (ImageJ)	Fiji	https://fiji.sc/
Mypic Zen autofocus macro	Jan Ellenberg	https://git.embl.org/grp-ellenberg/mypic
Graphpad Prism9	Graphpad	https://www.graphpad.com/
String GO terms identifier	String	https://string-db.org/
BioRender	–	https://BioRender.com

**Other**		

Alexa Fluor® 568 Phalloidin	Molecular Probes	A12380
DAPI	Sigma	D9542-5mg

### Experimental model and study participant details

#### Fly husbandry, genetics, and clone induction

All experiments were performed using *Drosophila* melanogaster fly strains maintained with standard husbandry techniques on Vienna standard cornmeal-molasses-agar media. All adult experiments were performed using female flies maintained at 25°C under a 12h light/dark cycle unless otherwise indicated. The flies' health and immune status were not assessed, and they were not subjected to any prior procedures. To identify regulators of the midline region formation, we performed an *in silico* screen based on data from the Bristle Screen Database,^26^ a genome-wide RNAi screen in the *Drosophila* notum. Candidate genes were knocked down using *UAS-RNAi* lines and two notum-specific Gal4 drivers (*pannier-Gal4* and *apterous-Gal4*). Adult thoraxes were imaged using a Leica S9i camera with a 5.5xGreenough Stereozoom and 1.6x objective. The notum thorax images displayed are representative pictures for each studied phenotype of adult female flies raised at 25°C. Midline width was quantified as the mean of three measurements taken between the most central bristle rows at anterior, middle, and posterior regions, normalized to the distance between the two posterior supra-alar (pSA) bristles.

### Method details

#### Immunohistochemistry

Wing imaginal discs were dissected from wandering third instar larvae in PBS on ice, fixed in 4% formaldehyde (30 min), and permeabilized in 0.4% PBT. F-actin was labelled with Phalloidin (1:500, Molecular Probes); nuclei were stained with DAPI (1 μg/mL, Sigma). Samples were mounted in Vectashield (Vector Labs) and imaged with a Zeiss LSM880 using 20x (NA 0.8) dry or 40x (NA 1.4) oil objectives.

#### Live imaging of the pupal notum and clone generation

To investigate the function of *hbs* and *rst*, RNAi clones were induced during early third instar using the FLP-out system in flies expressing either E-cadherin::GFP or miniCic::mScarlet. Clones were generated by applying a heat shock at 37°C for 15 min 48–72 hours prior to live imaging. White prepupae (0h after puparium formation, APF) were aged at 25 °C until 16–18h APF before dissection and imaging. For imaging of *UAS-ras*^*V12*^ and *UAS.egfr* clones, the cross and the progeny were kept at 18°C. To create clones, a 15min heat shock at 37°C was performed, 24–48 hours prior to live imaging. Prior to live imaging, the pupae were switched to 29°C for 8/9 hours for conditional activation (clones induced by the following lines: *hs-FLP22; hbs::EGFP; UAS-ras*^*V12*^ x *actin>y+> Gal4; 2xtub-Gal80*^*ts*^ *and hs-FLP22; hbs::EGFP; UAS-egfr* x *actin>y+> Gal4; 2xtub-Gal80*^*ts*^) and imaged at 29°C. For mounting, pupae were positioned between double-sided tape and coverslip spacers (assembled from four stacked #1 coverslips), dissected from the anterior end of the pupal case, and mounted in Halocarbon Oil 10S under a 20 × 40 mm #1.5 coverslip, sealed with nail polish.

#### Notum wounding

Laser-mediated tissue ablation was performed on an Andor Revolution XD equipped with a Yokogawa CSU-X1 spinning disk unit and a Andor Ultra 897 EMCCD camera using a Plan Apochromat 40×/1.3 NA oil-immersion objective. Ablation was carried using a micropoint system with an 365nm pulsed laser with. 440 coumarine dye at 80% laser power with a 16 Hz repetition rate and two consecutive repeats. The ablated area corresponded to a ∼400×400 px square (∼110×110 μm). Tissue deformation was imaged using the 488nm laser by acquiring z-stacks every 5 min. Images are Maximum intensity projections. Movies were bleach-corrected and background-subtracted using a rolling-ball radius of 50 px.

#### Image acquisition and cell elimination analysis

Live imaging was performed on a Zeiss LSM800 with fast Airyscan using a 40x oil objective (NA 1.4), acquiring Z-stacks (1 μm steps) every 5 min for 700 min (18–30h APF). Autofocus was maintained using E-cadherin::GFP plane as reference (Zen macro, Ellenberg Lab). Movies were captured near the scutellum, encompassing the midline and the aDC/pDC macrochaetae. Movies shown are maximum projections. Cell elimination was manually marked using the Cell Counter plugin in Fiji. Regions of interest (ROIs) were defined at movie onset based on the central bristle rows (midline) and the pDC bristle line (periphery) (Figure 1A). Only cells within each ROI during the entire recording were included in the analysis. Cell death probability was calculated as the ratio of extruded cells divided by the total initial cells per region (midline or periphery). Cells were considered “dying cells” if they or at least one of their daughter cells died before the end of the movie. Cell death quantification was not performed in regions posterior to the pDCs. Cell clones were manually segmented and identified in Fiji using cell contours defined by E-cadherin::GFP expression. The cell mixing index and apical cell area were quantified for control (*white* RNAi) and *hbs* knock-down clones at 18h APF. The cell mixing index was defined as the fraction of a cell’s perimeter that is shared with non-clonal (*wild-type*) neighbouring cells across the clone boundary and was calculated as the ratio between the boundary length shared with *wild-type* cells and the total perimeter of the clone (boundary length / perimeter). Cell apical area was measured in μm^2^ from cell contours in Fiji for both control and *hbs* knock-down clones.

#### PIV analysis

Tissue deformation was quantified using Particle Image Velocimetry (PIV) in MATLAB (PIVlab; has previously shown on.^11^^,^^12^ Two-pass analysis was performed with interrogation windows of 64 pixels (first pass) and 32 px (second pass), each with 50% overlap. Final vector field is composed of displacement vectors separated by 32 pixels (15.5 μm). Divergence was calculated on every 32px x 32px square and averaged over 240 or 700 min, as indicated in figure legends. Compaction rate (Convergence) is defined as ‘‘- Divergence’’ of the vector field (calculated on MATLAB). Averaged compaction rates were calculated by averaging on one PIV window the compaction rate over the full movie (700 min). Mild spatial smoothing in PIVlab (smoothing factor ≈0.33) was applied before computing divergence for the images shown in Figures 2L–2Q, and S3G–S3I, whereas no smoothing was applied for Figures S3E and S3F.

### Quantification and statistical analysis

Quantifications from adult thorax images are presented as mean ± standard deviation (SD). Normality was assessed using the Shapiro–Wilk test. Depending on the distribution, either parametric (unpaired t-test) or non-parametric (Mann–Whitney U) tests were applied. For live imaging-based cell death analysis, data are presented as mean ± standard error of the mean (SEM). To assess differences in binary cell fates (survival vs. death), statistical significance of cell elimination rates between conditions was determined using Fisher’s exact test, based on initial cell counts. Significance thresholds are indicated in the figure legends as ∗∗∗∗ p<0.0001; ∗∗∗0.0001> p>0.001, ∗∗0.001> p>0.01, ∗p0.01> p>0.05. All statistical analyses were performed using GraphPad Prism version 9.
