# Supplementary material for: Hbs and Rst adhesion molecules provide a regional code that regulates cell elimination during epithelial remodeling
Source: iScience. 2026 Feb 18;29(3):114971. doi: 10.1016/j.isci.2026.114971 (PMC12992536; doi:10.1016/j.isci.2026.114971)

iScience, Volume *29*

Supplemental information

Hbs and Rst adhesion molecules provide a regional code that regulates cell elimination during epithelial remodeling

Miguel Ferreira-Pinto, Mario Aguilar-Aragón, Christa Rhiner, and Eduardo Moreno

Figure S1 [Multi-step RNAi screen detailed results]

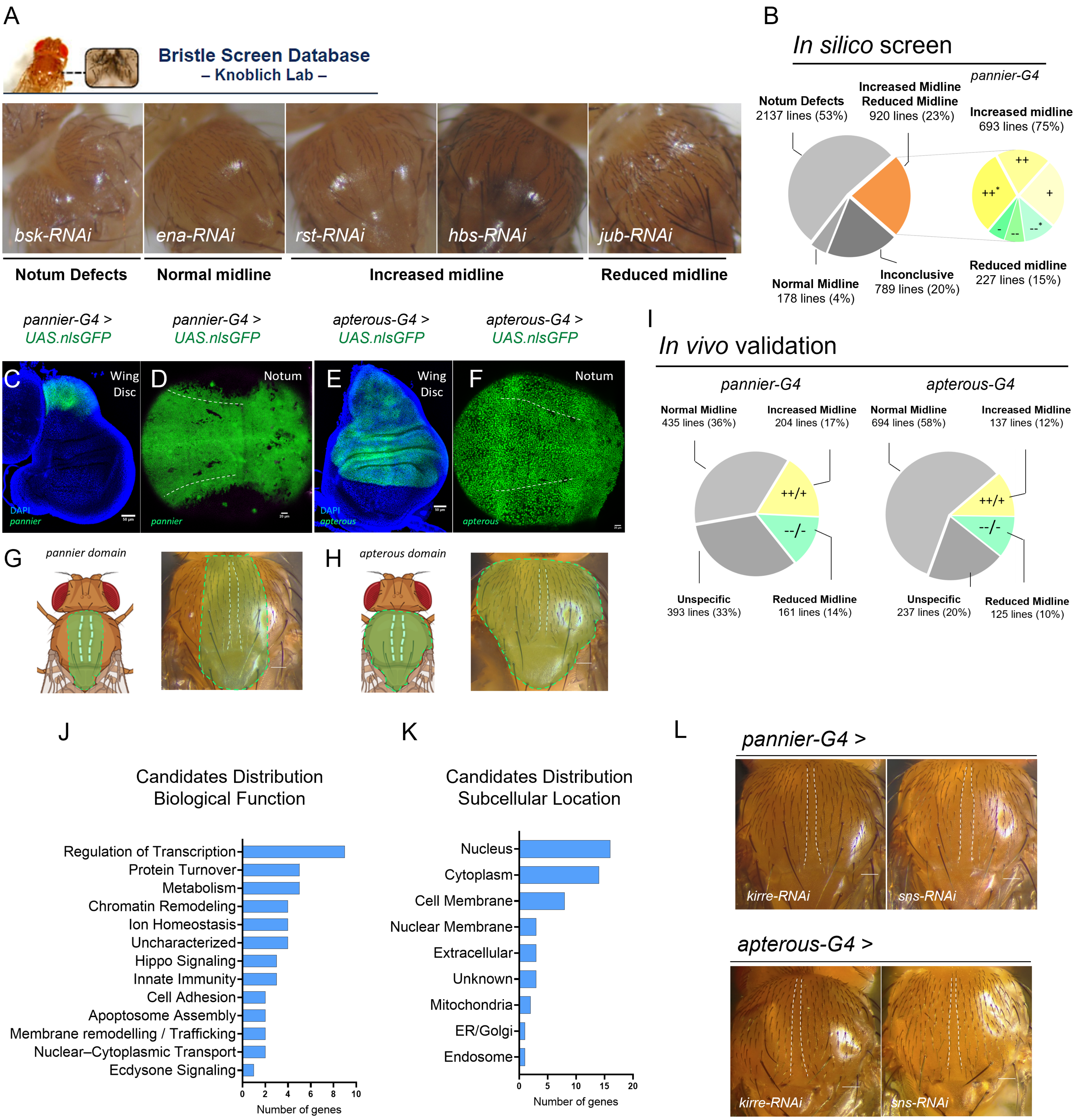

Figure S2 [Notum phenotypes of the 47 validated candidates from the screen]

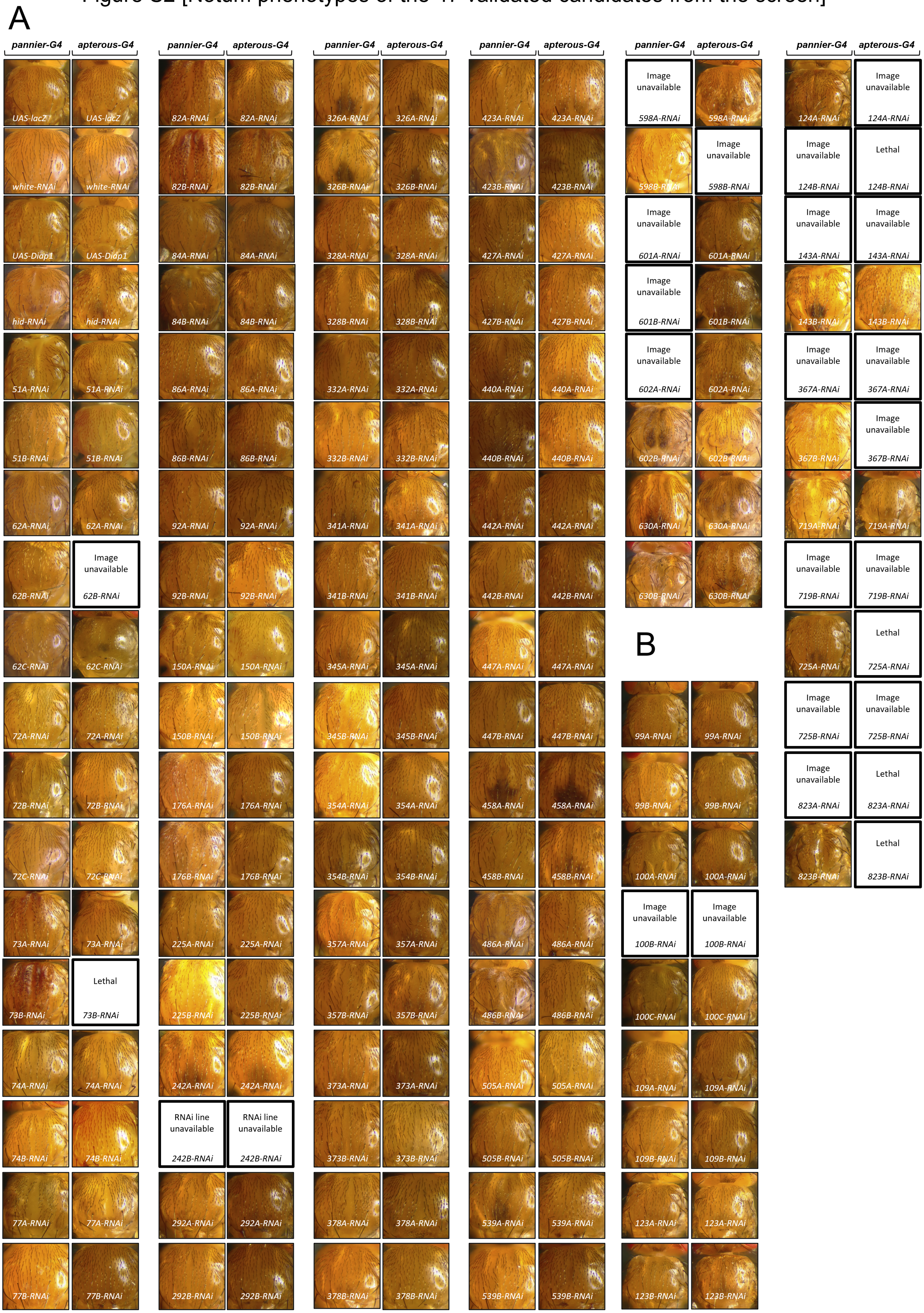

Figure S3 [Hbs and Rst expression pattern and their role on global and local epithelial remodelling.]

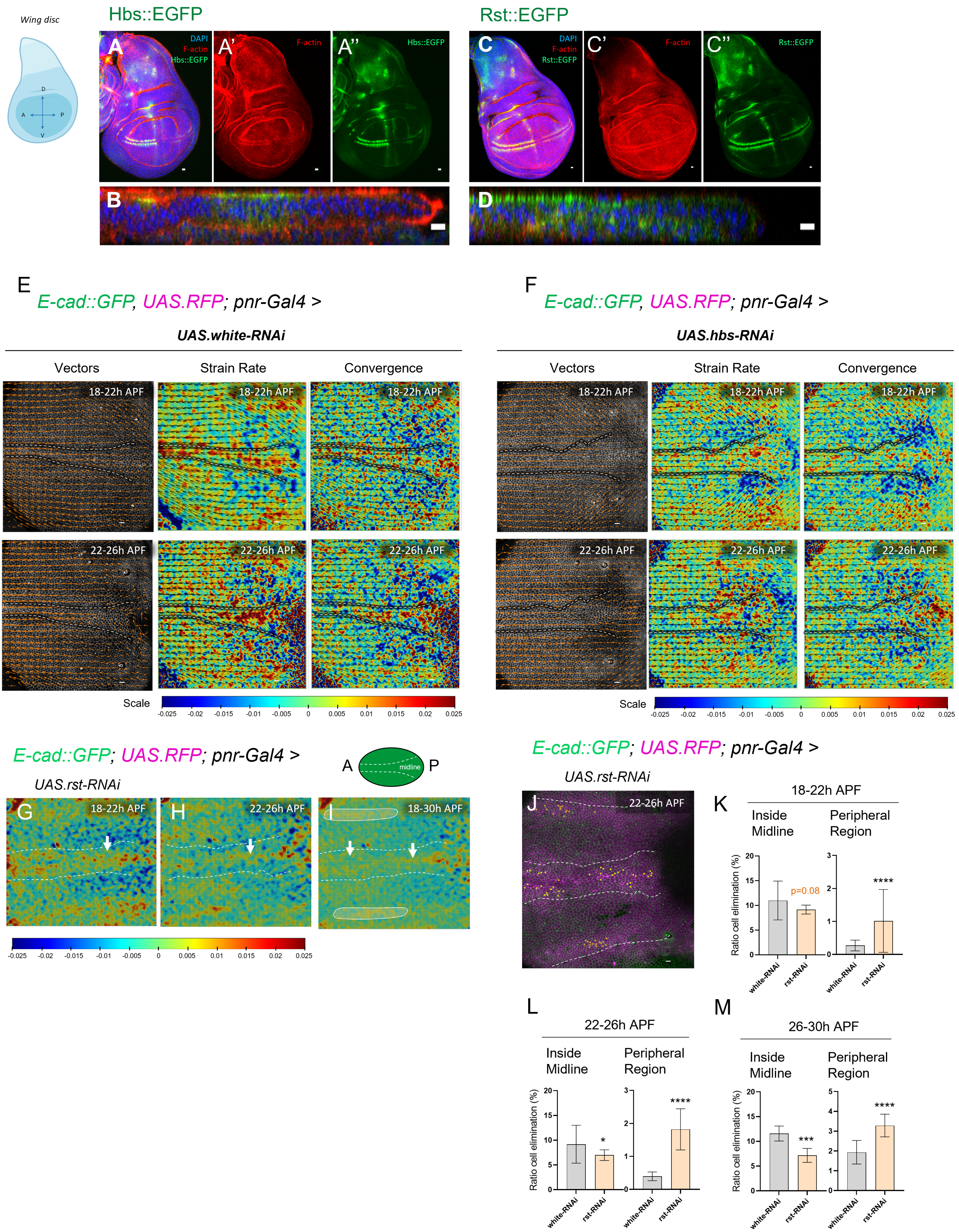

Figure S4 [Hbs and Rst expression patterns mostly correlate with EGFR/ERK activity but are differentially regulated by EGFR/ERK Signalling.]

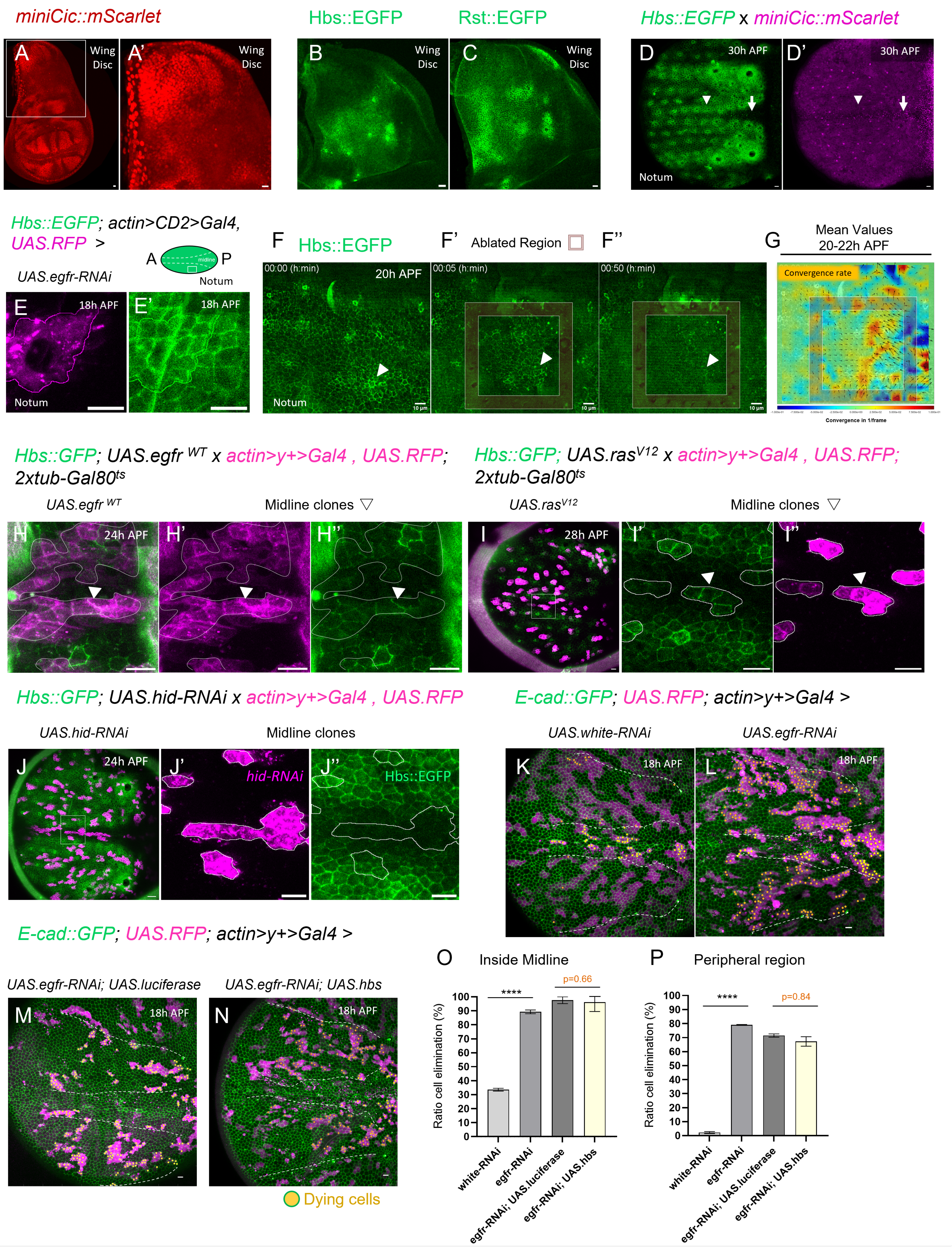

Supplement: Document S1. Figures S1–S4 [file mmc1.pdf]
